# Perianal fistulas: a new management approach using mesenchymal stem cells as a human, biological and autologous tool—a single-centre observational study

**DOI:** 10.1007/s00384-025-05020-7

**Published:** 2025-12-02

**Authors:** Alessandro Testa, Domitilla Passantino, Carlo Garbarino, Andrea Verdi, Tiziana Cozza, Domenico Mascagni, Chiara Eberspacher

**Affiliations:** 1 UOS Proctological and Pelvic Floor Surgery, S. Peter Hospital, Rome, Italy; 2 UOS Plastic Surgery, S. Peter Hospital, Rome, Italy; 3 Department of Surgery, “Sapienza” University of Rome, Viale Regina Elena 324, Rome, Italy

**Keywords:** Mesenchymal stem cells, Perianal fistulas, Surgery

## Abstract

**Purpose:**

The surgical treatment of perianal fistulas is challenging, especially in complex cases. Many surgical options may cause impairment of the anal sphincter with subsequent incontinence or may be less effective with a high number of relapses or the persistence of the pathology. New techniques, such as the use of mesenchymal stem cells, are becoming increasingly important because of their effectiveness and lower risk of complications.

**Methods:**

In this single-centre prospective observational study, patients with complex perianal fistulas were treated via the infiltration of mesenchymal stem cells that had been purified using the Lipogems® system.

**Results:**

This study included 63 patients with complex perianal fistulas who were treated with mesenchymal stem cells extracted from adipose tissue. Successful clinical healing was observed in 43 (68.25%) patients. Eleven (17.4%) patients presented recurrence after treatment, and 9 (14.3%) had persistent incomplete healing. Minor postoperative complications were observed in six (9.5%) patients, which were related to adipose tissue harvesting in three patients. In the majority of patients, postoperative pain was mild or not present.

**Conclusions:**

Mesenchymal stem cells offer an innovative therapeutic tool for treating perianal fistulas. This study confirms their safety and efficacy in treating complex perianal fistulas. Nevertheless, more extensive patient follow-up is necessary, as demonstrated by the most recent literature on related techniques.

**Supplementary Information:**

The online version contains supplementary material available at 10.1007/s00384-025-05020-7.

## Introduction

Perianal fistula is a common proctologic condition that requires surgical treatment. The incidence is approximately 1.69 per 10,000 individuals per year [1]. The goals of surgery to treat anal fistulas include safety and therapeutic efficacy as well as the use of minimally invasive and sphincter-saving methods [2]. Recently, new techniques and tools have been developed to improve surgical outcomes for complex perianal fistulas, specifically, fistulas that involve more than 30% of the external anal sphincter or that are associated with inflammatory bowel diseases, malignancies or treatment via irradiation [2]. Among these minimally invasive innovations, the ligation of the intersphincteric fistula tract (LIFT) and fistula laser closure (FiLaC) stand out, as they focus on the obliteration of the fistula tract mechanically or via a laser diode [3, 4]. Video-assisted anal fistula treatment (VAAFT), which involves the use of a fistuloscope to visualise and treat the fistula as well as the closure of the internal orifice, is another minimally invasive technique [5].

Further innovation involves the use of biomaterials to fill the lumina of fistula tracts and stimulate the formation of granulation tissue around fistulas. These materials are often derived from animal collagen or blood products and are provided in the form of plugs, pastes or glues [6, 7].

Following the introduction of biomaterials and the concept of regenerating fistulas by helping them heal through cellular migration, the idea of using mesenchymal stem cells (MSCs) was conceived. This new approach exploits adipose-derived MSCs, which infiltrate the fistula tract to close the lumen through a cell replication and differentiation process [8]. The infiltration of MSCs is particularly useful in the case of more challenging fistulas, such as those complicating Crohn’s disease [9]. The initially encouraging results derived from this method, which was applied to fistulas associated with inflammatory diseases, are the subject of discussion due to the decrease in success rates during the follow-up period [10]. Despite this, the development of devices to process human adipose tissue that contains a high number of activated MSCs without the use of enzymes or centrifugation has accelerated the use of this method in clinical practice [11]. In this study, we aimed to confirm the efficacy of the use of adipose-derived MSCs to heal complex perianal fistulas.

## Methods

We enrolled patients with trans-sphincteric perianal fistulas in a single-centre clinical trial. The study protocol was approved by the ethics committee and conducted in accordance with the 2008 Declaration of Helsinki [12]. Participants were recruited from July 2017 to March 2020 after they provided written informed consent. The inclusion criterion was the presence of complex perianal fistula in patients aged between 18 and 75 years. The definition of complex perianal fistula includes numerous typologies based on the American Gastroenterological Association classification: high fistulas that cross more than 30% of the external sphincter; fistulas with multiple external openings or separate tracts; anterior fistulas or associated rectovaginal fistulas in women; fistulas in patients with preexisting incontinence or specific risks, such as previous local irradiation; fistulas in patients with coexisting anal stricture; and fistulas in patients affected by Crohn’s disease [13]. The exclusion criteria included refusal to provide consent, acute perianal sepsis, proctitis, anorectal neoplasms or any type of cancer in the 12 months prior to surgery, pregnancy and major psychiatric disorders. All the patients underwent the following preoperative study protocol: proctologic examination with anoscopy, endoanal ultrasound or magnetic resonance imaging (MRI) of the pelvis to define the fistula tract(s) in relation to the sphincter anatomy and to identify any existing abscess collection, anorectal manometry to assess previous incontinence and colonoscopy if not already performed in the previous 2 years.

To ensure transparency and clarity in our reporting, our study adhered to the Strengthening the Reporting of Observational Studies in Epidemiology (STROBE) guidelines, which are particularly relevant for cohort studies [14]. We specifically followed the items related to study design (STROBE Items 4–8), presentation of results (STROBE Items 13–15) and interpretation (STROBE Items 18–21).

### Surgical technique

**Fig. 1:**
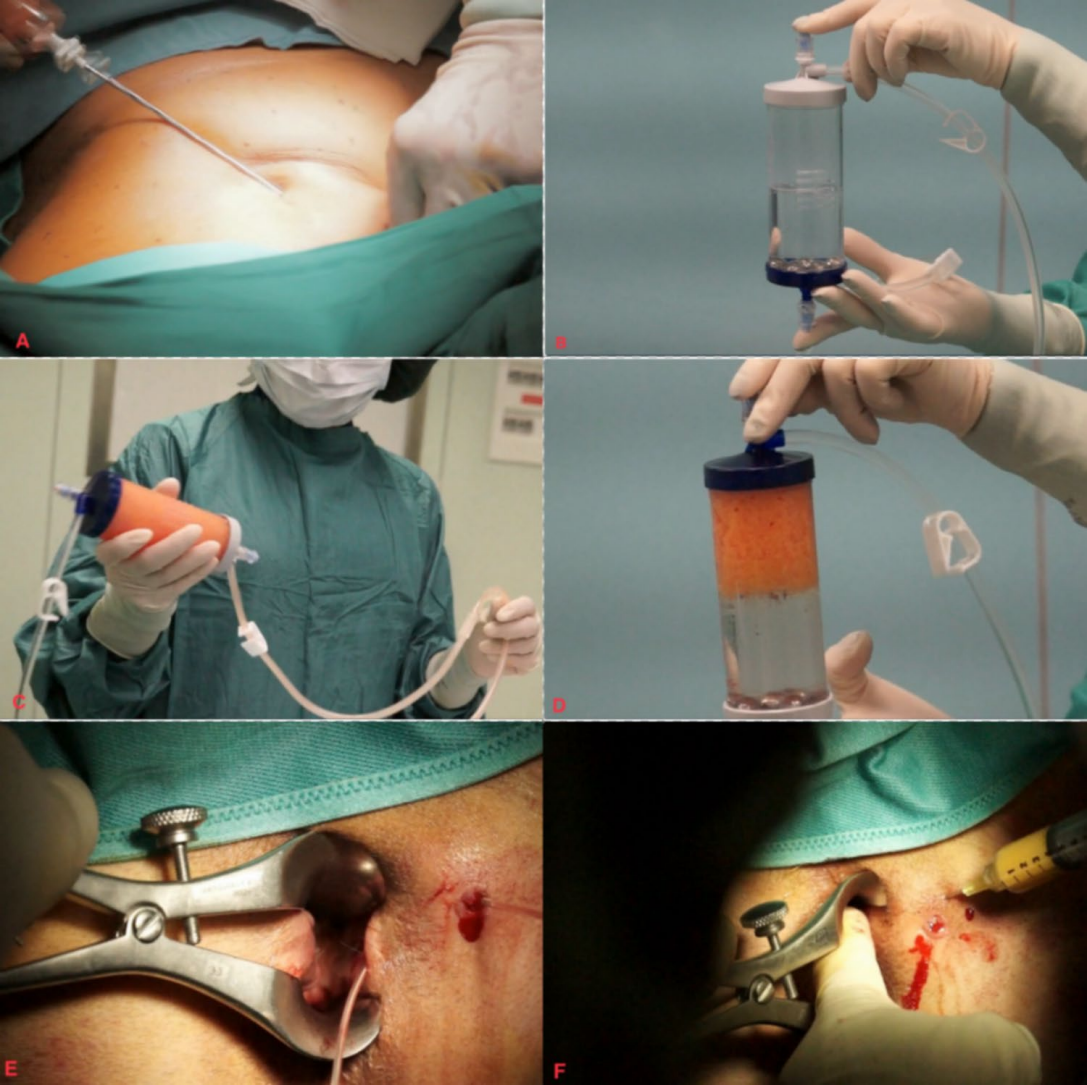
**A** Mini liposuction; **B**, **C**, **D** processing of the autologous adipose tissue with the Lipogems® system; **E** de-epithelisation of the fistula tract; **F** injection of the processed material into the entire area around the fistula

All the participants underwent the following surgical procedure (Fig. 1):

1. Mini liposuction of the middle abdominal quadrants. The procedure was performed with the patient in a supine position, first infiltrating with saline solution supplemented with lidocaine and epinephrine (Klein’s solution). The adipose tissue was aspirated with a 13 G blunt cannula connected to a syringe. The recommended volume of fat removed was at least 50 ml.
2. Intraoperative identification of the internal orifice and the entire fistula tract(s).
3. De-epithelisation of the fistula tract. This was performed with a ‘brusher’ or alternative method until near-complete removal of the epithelial lining and bleeding of the tract were achieved. In selected cases, a recently developed brusher called Fixcision, which can completely remove the epithelium lining the fistula wall, was used [15]. To allow its use, the fistula needed to have a straight tract trajectory and be no longer than 5 cm.
4. Suturing of the internal orifice after the scratching of its margins just behind the internal sphincter and the creation of a mucosal flap to exclude the fistula tract of the anorectal canal. This was performed with a 2/0 polyglycolic acid absorbable suture.
5. Processing of the autologous adipose tissue that was previously obtained through mini liposuction with the Lipogems® system to obtain material with a high concentration of pericytes and activated MSCs with high levels of regenerative activity.
6. Injection of the final purified MSCs into the entire area around the fistula, that is, all 360° relative to the tract path, from the deep mucosal level to the more superficial skin level.

All the patients underwent surgery in the knee-pectoral position (phases 2–6 of the surgical procedure) under general or spinal anaesthesia.

The Lipogems system [9, 16, 17] is a cylindrical device made of plastic material that contains medical steel balls and is connected through taps containing filter membranes to a continuous saline infusion system and a discharge system with a collection bag. The lipoaspirate is introduced into the device, which is already filled with saline, by connecting the syringe to an inlet tap, with the interposition of the appropriate filter. It then undergoes a ‘shaking’ action. This causes the beads to microfragment the adipose tissue, thereby separating it from dead cells, blood and oily debris, which are conveyed into the collection bag. Agglomerates or clusters of adipocytes that are approximately 200 microns in diameter are thus isolated in the device, and the vascular stromal units that are home to pericytes and activated mesenchymal cells, which represent the biologically active core of tissue regeneration, are enclosed. Their paracrine effect on host tissue promotes the healing process, which results in the obliteration of the tract and its orifices in 6–24 weeks [17, 18]. In the specific case of the Lipogems system, regulations allow its use in hospitals but with some procedural peculiarities. Its autologous characteristic must be maintained; thus, the entire procedure must be performed during the operating session, and the patient must have signed a clear informed consent form.

### Postoperative care

All the patients received 1 g of cefazolin and 500 mg of intravenous (IV) metronidazole when admitted to the operating room. Postoperatively, they were given IV paracetamol at 1000 mg (one vial × 2 until discharge) within 24 h after surgery. During the first week after discharge, the following home therapy was prescribed: oral metronidazole at 500 mg × 2 for 6 days, fibre (psyllium) to be taken in an aqueous solution and oral ketorolac at 20 mg as needed in the event of pain.

Checkups were scheduled on the seventh postoperative day and after 1 month, 3 months, 6 months and 1 year. The assessments during the checkup included a perianal and anorectal physical examination, with mucosal flap inspection; an evaluation of secretions (quantity and quality) and potential associated sepsis; an evaluation of the patency or closure of the external orifice; an evaluation of anal continence through the patient’s history and physical examination through digital rectal exploration; an evaluation of the patient’s quality of life via a questionnaire; and an evaluation of the resumption of work activities (timing and modalities). At the final checkup 1 year after surgery, a trans-anal ultrasound or pelvic MRI was performed.

The primary outcome of the study was the complete healing of the fistula and the absence of fistula drainage, with the closure of the treated external openings and the absence of abscesses. Failure was defined as a clinical diagnosis of fistula recurrence at any time during the postoperative follow-up period based on clinical interviews, physical examinations and trans-anal ultrasound or MRI. The secondary outcomes evaluated were postoperative pain, which was evaluated using a visual analogue scale (VAS) score; improvement in quality of life (improvement – no difference – worsening); satisfaction (patient very satisfied – satisfied – not satisfied); and complications. Continence was only clinically evaluated during the pre- and postoperative periods, so we did not report this as a clear outcome.

### Statistical analysis

The statistical analysis was performed with R version 4.1.0 software (R Core Team, Vienna, Austria). For the continuous variables, comparisons between the subgroups were made using Student’s *t* test or the Mann–Whitney *U* test depending on whether the distribution of the data was normal or nonnormal and checked using the Shapiro–Wilk test. Differences between the subgroups in terms of qualitative variables were investigated using Fisher’s exact test or a chi-square test. Logistic regression models to describe the associations between the response variables of interest and the main available explanatory variables were selected based on the Akaike information criterion. *p* values less than 0.05 were considered to indicate statistical significance.

## Results

### Patient characteristics

The present study included 80 patients with trans-sphincteric perianal fistulas who were followed prospectively. Seventeen patients dropped out of the study; thus, 63 were included in the final follow-up protocol. Their average age was 51.98 years (18–80 years), and 20 patients were female. Each participant had a complex perianal fistula. The fistulas were primary (49 patients) or recurring (14 patients), and all involved at least 30% external anal sphincter fibres. Seven patients had two fistula tracts originating from a single internal orifice. Of these, two had horseshoe fistulas. Four patients had Crohn’s disease and were being treated with mesalazine and corticosteroids but not biologic therapy at the time of the study. In 22 patients, a drainage loop was positioned in the fistula tract before surgery to ensure correct drainage and prevent abscess formation. Fifty-three patients underwent surgery under general anaesthesia, and 10 underwent surgery under spinal anaesthesia. All the patients’ baseline characteristics are presented in Table 1.

**Table 1:** Sociodemographic features, fistula characteristics and previous drainage loop positioning of the study population

Study population (*N*)	63
**Age** (years)^a^	51.98
**Sex**	
Male	43 (68.2%)
Female	20 (31.8%)
**Fistula characteristics**	
Primary	49 (77.8%)
Recurrent	14 (22.2%)
Single-tract fistula	56 (88.9%)
Multiple-tract fistula	7 (11.1%)
Horseshoe fistula	2 (3.2%)
Associated with Crohn’s disease	4 (6.3%)
Previous abscess	39(0.2%)
Rectovaginal fistula	1 (1.6%)
**Previous drainage loop positioning**	22 (34.9%)
**Follow-up (months ± standard deviation)**^**a**^	15 ± 3.05

Data are expressed as the number of patients and percentage

^a^Mean

The mean volume of material that was purified and then injected was 24 ml (range 5–40 ml). No intraoperative difficulties related to lipoaspiration or the use of the Lipogems system were encountered.

### Efficacy

Among patients treated only with MSCs, clinical success was achieved in 43 patients (68.25%), while 20 patients either did not achieve immediate clinical success (9/20) or experienced recurrence (11/20). Success was significantly associated with older age. The patients with successful clinical outcomes were, on average, older than those with treatment failure (54.2 ± 13.3 years vs. 41.4 ± 12.6 years, *p* = 0.008). Four patients with immediate treatment failure achieved complete healing after the infiltration of platelet-rich plasma (PRP). The success rate was higher among male patients (33/43, 76.7%) than among their female counterparts (10/20, 50.0%), which resulted in an odds ratio (OR) of 3.18 in favour of men, but this difference was not statistically significant (*p* = 0.088).

The same was true for the type of underlying condition, as the procedures for the patients with primary perianal fistulas (35/49, 71.4%) were successful more frequently than those with re-recurrent fistulas (8/14, 57.1%), but again, the difference was not statistically significant (*p* = 0.139). The presence of a drainage loop was also associated with procedural success (not significant; *p* = 0.079). Only 31 of 41 patients had successful outcomes without a drainage loop (75.6%), while success was observed in 21 of the 22 patients for whom a drainage loop was used (95.5%; OR, 6.62). No significant correlation (*p* = 0.091) was observed between the success of the procedure and the fistula type. Immediate healing was achieved in 37 of 56 patients with a trans-sphincteric fistula with a single tract compared with six of seven patients with a fistula with multiple tracts. A successful outcome was also achieved in the only patient with a rectovaginal fistula. In the four patients with fistulas associated with Crohn’s disease, success was achieved in three (75%); the only patient who did not respond to therapy immediately presented with symptoms indicative of a persistent fistula (Table 2).

**Table 2:** Efficacy of mesenchymal stem cell treatment for complex perianal fistulas

Patient characteristics		*n*	Primary healing		*p* value
			Yes (%)	No (%)	
*n*		63	43 (68.25)	20 (31.74)	
Sex	Male	43	33 (76.7)	10 (23.2)	0.088
	Female	20	10 (50)	10 (50)	
Primary fistula		49	35 (71.4)	14 (28.6)	0.139
Recurrent		14	8 (57.1)	6 (42.9)	
Single-tract fistula		56	37 (66.1)	19 (34)	0.091
Multiple-tract fistula		7	6 (85.7)	1 (14.3)	
Crohn’s disease	Yes	4	3 (75)	1 (25)	
	No	59	40 (67.8)	19 (32.2)	

**Fig. 2:**
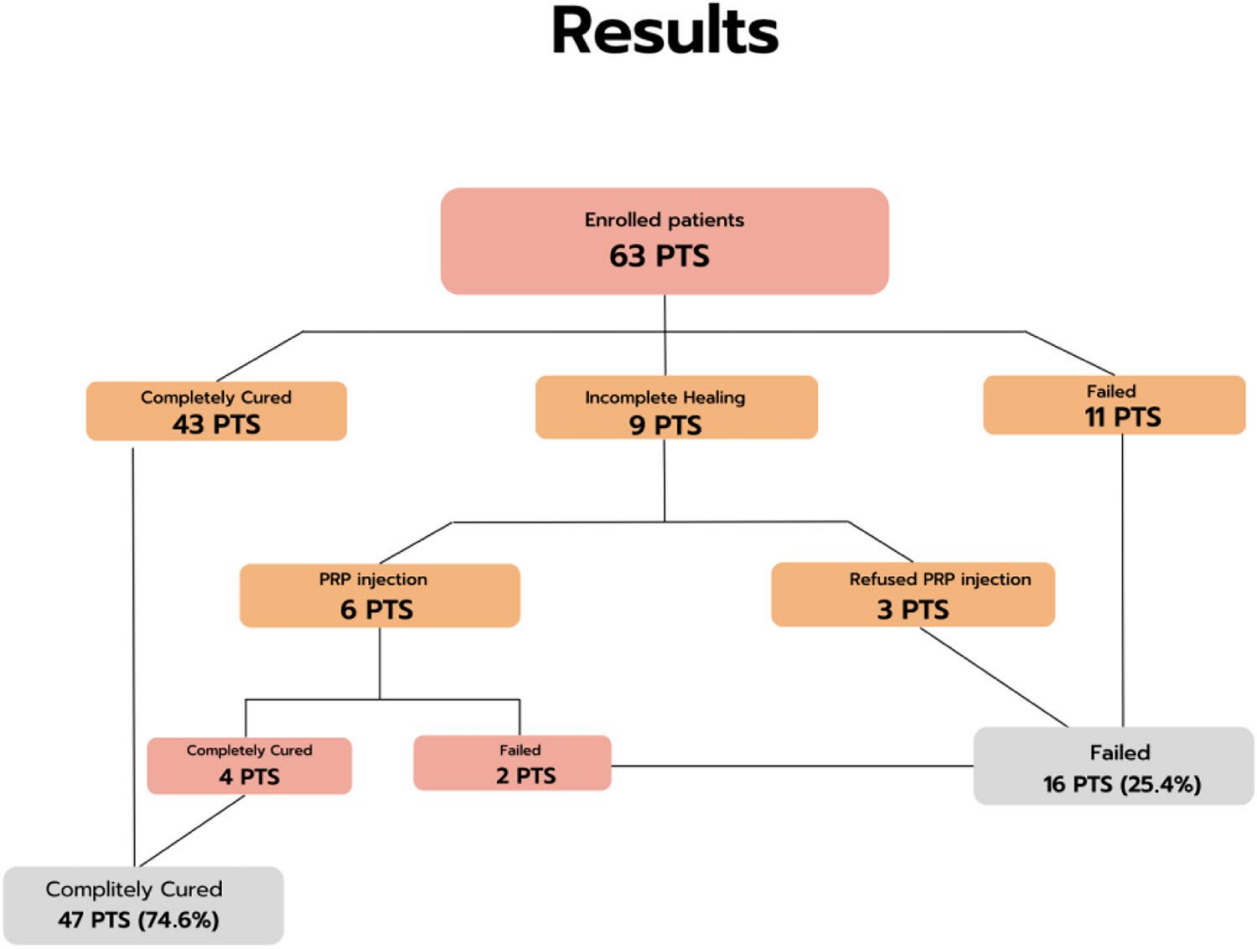
Results for the group of 63 patients who were enrolled and monitored for at least 12 months

Sixty-three patients were treated and monitored for at least 12 months (Fig. 2). The average follow-up period was 15 months. Success was observed in 43 patients (68.25%) who were completely cured from a clinical standpoint, i.e. in outpatient evaluations based on the parameters established for the follow-up protocol. In nine patients, healing was regarded as incomplete, i.e. closure of the internal orifice but without complete obliteration of the fistula tract, in some patients even 2-mm distal. Among these nine patients, six underwent outpatient treatment with a PRP injection [19] into the residual tract. At a further follow-up visit after 6 months, four patients were considered completely cured when reassessed, while two were not. Three patients refused the recommended treatment. Eleven patients who were completely cured with MSCs experienced recurrence, as did two of the patients who underwent outpatient PRP treatment.

The time required for the closure of the external orifice was highly variable and unpredictable. The timeframe ranged from 5 weeks to 6 months after the surgical procedure.

### Secondary outcomes and complications

In most of the patients, postoperative pain, as evaluated using the VAS at the first checkup on the seventh postoperative day, was mild (17 patients) or not present (39 patients). Almost all the patients who reported moderate (five patients) or severe pain (two patients) referred to it as being located at the liposuction site, although one patient reported anal pain.

None of the patients reported intraoperative complications. Postoperative complications were observed in six of the 63 patients (9.5%). Of these, three were related to adipose tissue harvesting (haematoma and abdominal wall pain), one to anal pain, one to fever and one to perianal abscess. No statistically significant relationships were observed between patient characteristics and complications for any of the variables analysed, i.e. sex, age, prior intervention, previous abscess, type of fistula, underlying condition or volume of Lipogems.

**Fig. 3:**
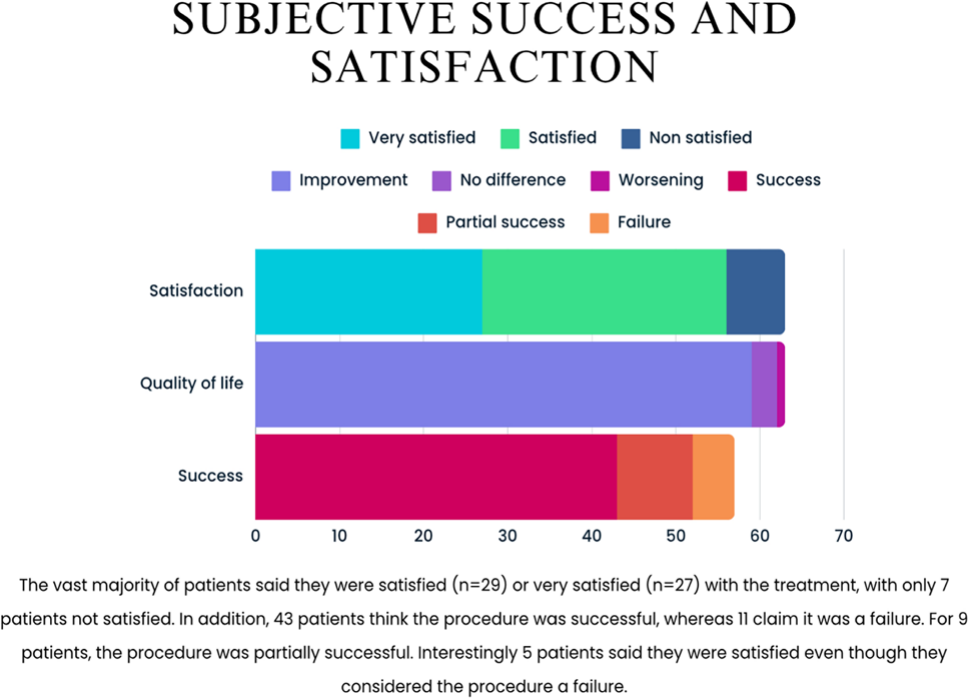
Patients’ satisfaction and quality of life improvement after MSC treatment

Continence was clinically evaluated during the follow-up period, and no significant difference was observed between the pre- and postoperative periods. The vast majority of the patients reported that they were satisfied (*n* = 29) or very satisfied (*n* = 27) with the treatment, with only seven patients indicating that they were not satisfied. In addition, 43 patients reported that the procedure was successful, whereas 11 reported that it was a failure. For nine patients, the procedure was partially successful. Notably, five patients reported that they were satisfied even though they considered the procedure a failure (Fig. 3). Satisfaction did not correlate with any specific parameters, whereas subjective success was significantly associated with older age (*p* = 0.047) and a lower volume of Lipogems being used (*p* = 0.013). Subjective success was also associated with male sex, although in this case, the significance threshold was not reached (*p* = 0.054). With respect to perceived quality of life, only four patients reported no improvement after treatment (one described worsening, and three reported no difference). All four patients also indicated that they were not satisfied.

## Discussion

Treating complex perianal fistulas with a minimally invasive technique, both in the ‘demolishing’ and ‘reconstructive’ phases, is a major goal of proctologic surgery. Some methods are more traditional, e.g. setons, fistulotomies and fistulectomies with or without sphincteroplasty, and can impair continence due to sphincter damage, whereas other techniques, such as LIFT, minimise incontinence without the use of a particular device [3, 20]. Methods such as FiLaC and VAAFT, which leverage technological and instrumental innovations, have also recently been proposed [4, 5]. The costs of such methods are higher, but the outcomes are satisfactory even if the number of relapses may be greater. Another technique that is often associated with the closure of the internal orifice is the obliteration of the fistula tract with various materials, such as cones or plugs made of a collagen matrix or specific glues [6, 7]. Although this is interesting from a technical perspective, such techniques nevertheless have several drawbacks, such as plug dislocation due to mispositioning or the rejection of the material used. These materials are generally biological, although synthetic materials also exist [21].

The idea of using a biological tool in a minimally invasive, sphincter-saving procedure with no adverse events and a quick and comfortable postoperative period has received immense support. The study of the use of adipose tissue mesenchymal cells in tissue regeneration and their application to perianal fistulas has represented a turning point in attempts to overcome the limitations of other sphincter-saving techniques. Initial studies focused on the use of allogeneic, expanded adipose-derived stem cells; after expansion according to cell culture practices, these cells were cryopreserved until use [22]. The studies of Garcia Olmo et al. [9, 10, 23], Ciccocioppo’s group [24, 25] and Caplan at Cleveland University [26] have helped disseminate these methods, which have shown to be quite effective, especially compared to the other available procedures. In recent years, the possibility of using new devices to extract and purify autologous MSCs from patients with anal fistulas has changed the game: the tissue is not taken from donors, compatibility does not need to be assessed and MSCs do not need to be expanded or cryopreserved. This has made the use of mesenchymal cells more manageable in hospital settings [27]. As described in the “Methods” section, we used the Lipogems system to obtain MSCs. The curettage of the fistula tract and the debridement of the internal opening remain essential, as they are widely believed to minimise the risk of recurrence and allow for the correct regeneration of the surrounding healthy tissues [28]. The suturing of the internal orifice, which is behind the sphincter fibres, is almost mandatory, sometimes with a mucosal flap above the suture of the internal orifice to further support its closure. The use of techniques to close the internal orifice, combined with the use of MSCs, could be considered by some to be the cornerstone of fistula healing. We have used a mucosal flap, which yields good results in terms of fistula healing on its own. However, there is certainly no evidence showing whether the closure of internal orifices influences healing more than the use of mesenchymal cells, as has not been demonstrated in other techniques that combine sphincter-sparing methods with internal orifice closure [29].

In this study, we evaluated the success of fistula healing by evaluating many factors, such as the quantity and quality of secretions during the postoperative period, signs of local sepsis, the presence of fever that could not be ascribed to any other cause, and local pain and symptoms resulting from liposuction, e.g. ecchymosis, haematoma. Based on our experience with these first cases, we believe that 6 months is the period required for recovery, although to consider such a condition resolved, stable quiescence must be observed for at least an additional 6 months. When healing can be confirmed through instrumental procedures, endoanal ultrasound scans or pelvic MRI with contrast, it can then be classified as ‘combined healing’ [30].

The aim of our study was to preliminarily determine the efficacy of MSC therapy for complex perianal fistulas in a single-centre study. With a healing rate of 68.25% and a minimum follow-up of 12 months, the results indicated that MSC infiltration was an effective treatment method for perianal fistulas. Our data agree with the initial results of a study in Spain, which addressed fistulas related to inflammatory disease only [10]. Even in Naldini’s study on cryptoglandular fistulas, similar healing data (57–83%) were observed [28].

Complications are rare and are often linked to the site from which the cells were taken rather than the area in which the infiltration occurred. Even more importantly, patient satisfaction with the procedure itself, which appears to combine efficacy with microinvasiveness, is high [8]. Nevertheless, the publication of the follow-up results of the Phase 3 ADMIRE-CD study has dampened enthusiasm: at week 104, clinical remission was reported in 14/25 (56%) patients in the group treated with MSCs vs. 40% in the control group [10]. In scientific discussions about the study data, it emerged that the response rate of the placebo group (with fistula preconditioning) was significant and not dissimilar to that of the group for which MSCs were used [31]. Despite the less encouraging reflections on this procedure after these results, the utilisation of MSCs for perianal fistulas must not be abandoned: only a few patients were followed for such a long time in the ADMIRE-CD study, and it is not possible to make generalisations. Furthermore, the fistulas that were treated were associated with inflammatory bowel disease, and no equivalent study analysing the application of MSCs in cryptoglandular fistulas has been conducted.

Our study has several limitations that could be addressed in future research. This single-centre study did not involve any randomisation of the patients, and a control group was not included. Furthermore, the sample size was small, which reduces the generalisability of the results. While the initial group of patients showed promising outcomes, many were lost to follow-up and were therefore not included in the study, which limited our analysis. Prospective data collection was performed, but the lack of a standardised and rigorous evaluation of continence and patient satisfaction levels via a quality of life questionnaire deprived the study of outcomes that could be compared in future studies. It would be useful to study new cases and carry out trials with larger samples and control groups to corroborate our results, which, at present, seem encouraging.

## Conclusions

Mesenchymal cells, which are derived from human autologous adipose tissue, represent an innovative therapeutic tool to treat perianal fistulas and can be used as an alternative to demolishing surgery. In this study, we showed that perianal fistula treatment with MSCs is a safe procedure that has acceptable recurrence rates and preserves anorectal function and continence. The procedure did not involve any particular complications and was well accepted by the patients in this study. The follow-up showed that the duration of healing was quite long, but the patients experienced no particular discomfort. Additional studies with considerably larger participant numbers and control groups are necessary to confirm the efficacy of MSCs in the treatment of perianal fistulas.

## Supplementary Information

Below is the link to the electronic supplementary material.

ESM 1 (DOCX 30.4 KB)

## Data Availability

The data that support the findings of this study are not openly available due to reasons of sensitivity and are available from the first author upon reasonable request. The data are stored in a controlled access database at San Pietro Hospital.
